# Clonal hematopoiesis in primary immune thrombocytopenia

**DOI:** 10.1038/s41408-022-00641-5

**Published:** 2022-03-15

**Authors:** Yanming Wang, Tianshu Yu, Qiaofeng Dong, Shuang Liu, Yafei Yu, Hong Yu Zhao, Ji Ma, Lin Dong, Liang Wang, Daoxin Ma, Yajing Zhao, Yu Hou, Xinguang Liu, Jun Peng, Ming Hou

**Affiliations:** 1 Department of Hematology, Yantai Yuhuangding Hospital Affiliated to Qingdao University, Yantai, China; 2 Department of Hematology, Qilu Hospital, Cheeloo College of Medicine, Shandong University, Jinan, China; 3 Department of Hematology, Heze Municipal Hospital, Heze, China; 4 Department of Hematology, Taian Central Hospital, Taian, China; 5 Department of Hematology, Jinan Central Hospital Affiliated to Shandong University, Jinan, China; 6 Department of Medical Oncology, Shandong Cancer Hospital and Institute, Shandong First Medical University and Shandong Academy of Medical Sciences, Jinan, Shandong Province China; 7 Department of Hematology, The First Affiliated Hospital of Shandong First Medical University, Taian, China; 8 Department of Hematology, Shengli Oilfield Central Hospital, Dongying, China; 9 Shandong Provincial Key Laboratory of Immunohaematology, Qilu Hospital, Cheeloo College of Medicine, Shandong University, Jinan, China; 10 Leading Research Group of Scientific Innovation, Department of Science and Technology of Shandong Province, Qilu Hospital, Cheeloo College of Medicine, Shandong University, Jinan, China; 11 Key Laboratory of Cardiovascular Remodeling and Function Research, Chinese Ministry of Education and Chinese Ministry of Health, Qilu Hospital, Cheeloo College of Medicine, Shandong University, Jinan, China

**Keywords:** Haematological diseases, Autoimmune diseases

Primary immune thrombocytopenia (ITP) is an autoimmune disorder characterized by decreased platelet count and increased risk of bleeding. Clonal hematopoiesis (CH) is described as the expansion of a clonal blood cell population with somatic mutations. It was first raised in a study of X-chromosome inactivation among elderly healthy women [1], and has been extensively identified in various clinical settings as the wide use of high-throughput sequencing technologies [2, 3]. However, the association between CH and ITP remains unclear. The diagnosis of ITP needs to exclude other thrombocytopenic conditions, such as hypoplastic myelodysplastic syndrome (hMDS) and inherited thrombocytopenia, which might be misdiagnosed as refractory ITP. [4] The updated international consensus report recommended the conduction of targeted gene sequencing to assess the genes associated with clonal malignancy in elderly ITP patients or those not responding to the initial treatments [5]. In the present study, the prevalence of CH was 10.78% in ITP, which was lower than in aplastic anemia (AA) and hMDS. Moreover, CH in ITP showed a close relationship to disease severity and treatment responsiveness.

We collected peripheral blood or bone marrow samples from 105 ITP patients (56 females and 49 males), 126 AA patients (50 females and 76 males), and 80 hMDS patients (28 females and 52 males) between January 2016 and December 2021 in eight hospitals. DNA was extracted from peripheral blood or bone marrow mononuclear cells of all patients by using MagMAX™ DNA Multi-Sample Ultra 2.0 Kit (Applied Biosystems™). Follow-up was initiated at the date of sample collection and ended in December 2021 or at the date of death. All patients fulfilled the diagnostic criteria established by the Chinese Society of Hematology [6–8], including history, physical examination, peripheral blood count and smear, and bone marrow examination consistent with ITP. Assays for plasma anti-glycoprotein (GP) autoantibodies and thrombopoietin levels were also carried out in complex and difficult cases. The definition of hMDS is described as previously [9]. The duration of ITP ranged from less than 1 week to 29 years, including 45 newly diagnosed patients and 60 persistent/chronic patients. Our study was approved by the ethics committees of all hospitals with informed consent from all patients.

To detect the CH, we designed a custom sequencing panel based on custom NovaSeq^TM^. The panel covered the gene mutations that were commonly observed in patients with MDS, acute myelogenous leukemia, and AA. Coding regions of 38 genes and/or mutational hotspots were sequenced (Supplemental Table 1). The panel consisted of 370 amplicons with a 300 bp base-pair (designed by AmpliSeq Design Studio). The sequence was executed on an illumina MiseqDx PE250 platform with 90% coverage. Bases of sequence with Phred score *Q* < 20 and population variants whose frequencies exceeded 1% were ruled out. The variants were annotated by the Ensembl GRch38. The detection threshold for mutations was determined as the variant allele frequency (VAF) ≥ 1%, and the cutoff value for defining CH was VAF ≥ 2%, whereas VAF > 40% were discarded for the possibility of a germline variant. Kolmogorov–Smirnov test, Student’s *t* test, Mann–Whitney test, Chi-squared test, logistic regression, and Pearson correlation analysis was used to analyze the results. Statistical analysis was performed using SPSS 24.0. *P* values < 0.05 were considered statistically significant, data were presented as mean ± SD or median and range.

It was reported that the misdiagnosis rate was ~14% in ITP [4]. In the present study, 13 ITP patients were found to harbor CH at first, among which two cases were misdiagnosed. The first case was a 53-year-old man with skin ecchymosis for 6 months. He was resistant to the first- and second-line therapies and was finally diagnosed with MDS at 4th-year follow-up. The other misdiagnosed patient was a 31-year-old female with menorrhagia and isolated thrombocytopenia, refractory to corticosteroids and other ITP-specific treatments, and diagnosed with congenital thrombocytopenia after re-evaluation of the disease. There were two misdiagnosed patients in non-CH group, who eventually turned out to be acute myelogenous leukemia and large granular lymphocytic leukemia, respectively. The four cases were excluded in the following analysis. In addition, three ITP patients with mutations (CEBPA, DNMT3A, and SF3B1) were not included in the analysis because the VAF values were <2% (Table 1 and Supplemental Table 2).

**Table 1:** Clinical characteristics of CH-ITP patients (VAF ≥ 2%) and patients with low-burden mutations (1% < VAF < 2%).

Patient ID	Age	Sex	Smoking history	Platelet count (×10^9^/L)	Disease duration (months)	Gene	VAF	Malignant transformation	Previous/present treatments	Initial response to corticosteroid	Refractory ITP	Bleeding symptoms	Bleeding score
ITP1	71	M	Yes	7	6	DNMT3A	0.0466	No	Dex, Pred, Rit, TPO-RA	NR	Yes	EP EC	S1M1
ITP2	72	F	No	11	1.5	ASXL1	0.0539	No	Dex, rhTPO,Rit, TPO-RA	NR	Yes	EC GH	S1M1
ITP3	31	F	No	1	72	DNMT3A	0.0454	No	Dex. IVIg, Rit, TPO-RA, Tacrolimus	NR	Yes	EC GI GYN ICH	S1O2 (intracranial3)
ITP4	62	F	No	13	8	DNMT3A	0.0692	No	Dex	R	No	PT	S1
ITP5	45	F	No	7	1	TP53	0.0739	No	Dex, Chinese medicine	CR	No	EC	S1
ITP6	84	M	No	19	24	SRSF2	0.0588	No	Pred, Dex	R	No	EC	S1
ITP7	84	M	Yes	26	0.5	ASXL1	0.0945	No	Pred, rhTPO	NR	No	No	0
ITP8	50	M	Yes	5	48	DNMT3A	0.0290	No	IVIg	-	No	EC GI	S1O2
ITP9	69	M	Yes	4	2.5	TET2	0.0625	No	Pred, TPO-RA	R	No	GH	M1
ITP10	56	F	No	14	12	DNMT3A	0.0313	No	Dex, IVIG, rhTPO, Rit	NR	Yes	GH EP	M2
ITP11	67	F	No	1	0.1	DNMT3A TET2	0.0540 0.0580	No	Pred	CR	No	GH EP GUH	M3
ITP12	54	M	No	6	348	CEBPA	0.0125	No	Pred, rhTPO, Spl	NR	Yes	EC GH	S1M1
ITP13	67	M	No	8	132	DNMT3A	0.0171	No	Dex, Pred	CR	No	No	0
ITP14	50	M	Yes	3	180	SF3B1	0.0129	No	Pred, CsA, IL-11, rhTPO, TPO-RA, Rit	R	Yes	GUH GH EP	M2O2

*M* male, *F* female, *PT* petechiae, *EC* ecchymoses, *EP* epistaxis, *GUH* genitourinary hemorrhage, *GH* gingival hemorrhage, *GI* gastrointestinal hemorrhage, *GYN* gynecologic hemorrhage, *ICH* intracranial hemorrhage, *HP* hemoptysis, *Dex* dexamethasone, *IVIg* intravenous gamma globulin, *Pred* prednisone, *Spl* splenectomy, *TPO* thrombopoietin, *TPO-RA* thrombopoietin receptor agonist, *Rit* rituximab, *CsA* cyclosporin A, *CR* complete response (platelet count ≥ 100 × 10^9^/L and absence of bleeding), *R* response (platelet count ≥ 30 × 10^9^/L and at least twofold increase of the baseline count without bleeding), *NR* no response (platelet count <30 × 10^9^/L or less than twofold increase of the baseline platelet count or bleeding).

Bleeding manifestations were grouped into three major domains according to ITP-specific Bleeding Assessment Tool: skin (S), visible mucosae (M), and organs (O), with a gradation of severity (SMOG). Severe bleeding was regarded as grade 3 for skin, and/or grade 2 or higher for mucosal domains, and/or higher than grade 1 for organ domain (S ≥ 3 and/or M ≥ 2 and/or O > 1).

Based on the present data, there was no significant difference in age between ITP patients and AA/hMDS patients (Fig. 1A). The prevalence of CH was calculated after adjusting age and sex. ITP patients had significantly lower prevalence (10.78%) than AA (19.05%, OR 5.99, 95% CI 5.01–7.16, *P* < 0.001) and hMDS patients (68.75%, OR 20.34, 95% CI 16.71–24.77, *P* < 0.001; Fig. 1B). The VAF value of mutations in ITP was also much lower than that in AA (5.64% ± 1.80% vs. 12.46% ± 8.27%, *P* = 0.008) and hMDS (5.64% ± 1.80% vs. 16.41% ± 11.54%, *P* = 0.002; Fig. 1C). In patients with CH, 16.7% AA cases and 23.6% hMDS cases had two or more mutations while only one ITP patient had more than one mutation (Fig. 1D). Gender was not found to be the influencing factor of CH in our study (Fig. 1E), whereas aging was indeed a promoter in the occurrence of CH in ITP (Fig. 1F). No significant association was observed between VAF and age in ITP patients with CH (Fig. 1G). There was no obvious correlation between CH in ITP patients and several chronic conditions including disease duration, hypertension, diabetes, anti-platelet glycoprotein autoantibodies, cerebral infarction, and coronary heart disease (Supplemental Table 3). Moreover, no difference was observed in blood cell indices between CH-harboring group and the non-CH group in ITP patients (Supplemental Table 4).

**Fig. 1:**
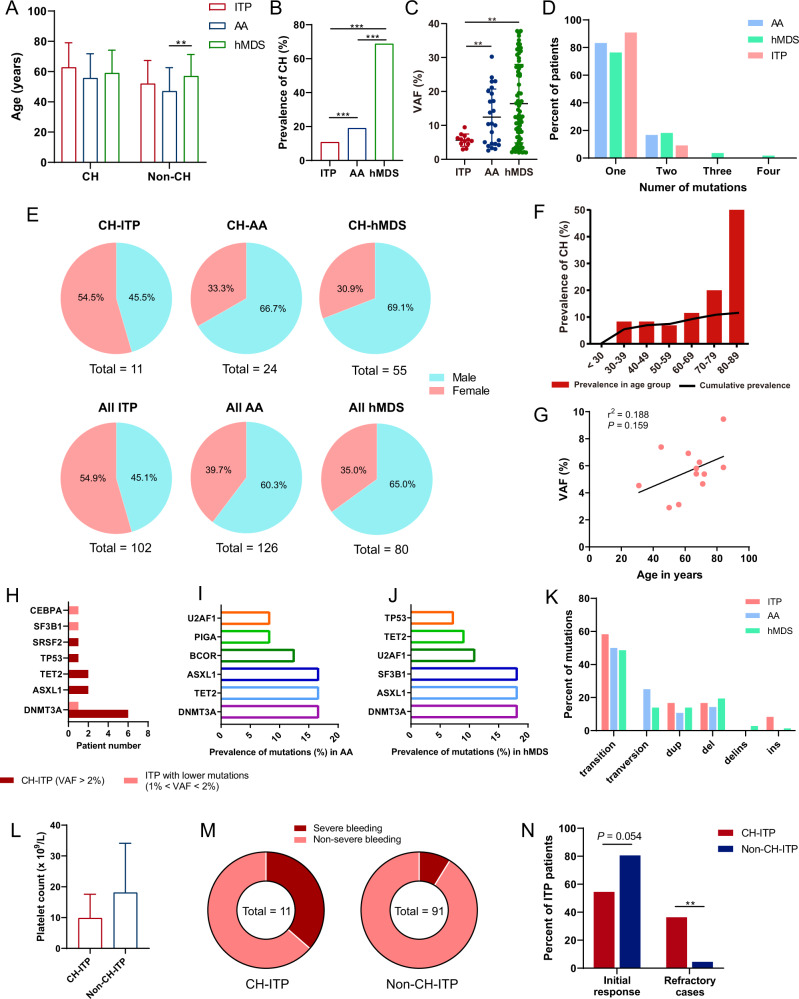
CH in ITP, AA, and hMDS patients. **A** Age profile of patients with ITP, AA, and MDS. **B** The prevalence of CH in ITP was much lower than that in AA and hMDS. **C** The VAF value of mutations in ITP patients was much lower than that in AA and hMDS. **D** Mutation number distribution in all CH patients. **E** The gender ratio of patients with ITP, AA, and hMDS. **F** The prevalence of CH in ITP patients over 65 years old was higher than that under 65 years old (27.27% vs. 6.25%, *P* = 0.005). The polyline represents the cumulative prevalence of CH in ITP patients, the bars show the percentage of patients with CH in different age groups. **G** No statistically significant correlation was found between mutation VAF and age in ITP patients. **H** The prevalence of different genes in ITP patients with CH or with low-burden mutations. **I** The top six most frequently mutated genes in AA patients with CH. **J** The top six most frequently mutated genes in hMDS patients with CH. **K** The percentages of different mutation types in all mutations. **L** There was no statistical difference in the platelet count between CH and non-CH-ITP patients (9.82 ± 7.77 vs. 18.07 ± 16.04, *P* = 0.097). **M** Severe bleeding cases occurred in 36.4% CH-ITP patients and 8.8% non-CH-ITP patients. The platelet count and patient age were included in the confounders in the analysis. **N** The initial response rate in patients with CH was lower compared with patients without CH though it did not reach statistical significance. The proportion of refractory cases in patients with CH was much higher than that in non-CH patients. All data analyzed by *t* test were normally distributed and similar in variance. CH clonal hematopoiesis, VAF variant allele frequency, del deletion, dup duplication, ins insertion, delins deletion-insertion.

Mutations of the genes involved in the regulation of DNA methylation accounted for the majority of acquired mutations in healthy individuals [10]. Accordingly, mutations of DNMT3A were the most common among ITP, AA, and hMDS (Fig. 1H–J, Supplementary table 2, 5 and 6), and most of the CH in our study tended to be driver mutations in hematologic cancers [11]. In ITP group, more than half of the patients had mutations of DNMT3A, followed by ASXL1/TET2 (18.2%) and SRSF2/TP53 (9.1%). By contrast, the most frequently mutated genes were DNMT3A/ASXL1/TET2 (16.67%), BCOR (12.50%), and PIGA/U2AF1 (8.33%) in AA patients. Similarly, gene mutations in hMDS were concentrated in DNMT3A/ASXL1/SF3B1 (18.18%), U2AF1 (10.91%), TET2 (9.09%), and TP53 (7.27%). Single-nucleotide variants were dominant in all types of mutations, among which transitions were the most prevalent (Fig. 1K).

The occurrence of CH in ITP is closely related to clinical symptoms and treatment prognosis. The bleeding severity was evaluated according to the ITP-specific Bleeding Assessment Tool [12]. Although the platelet count between CH and non-CH-ITP patients was not statistically different (Fig. 1L), CH was positively correlated with bleeding severity as ITP patients with CH were 6.95 times more likely to develop severe bleeding than those without CH (*P* = 0.008, Fig. 1M and Table 1). Corticosteroids are the first-line therapy for ITP with the overall response rates ranging from 70% to 80% [13]. A total of 87 patients received corticosteroid treatments in the present study. The initial response rate in patients with CH was lower compared with patients without CH (54.5% vs. 80.6%, *P* = 0.054, Fig. 1N), suggesting that ITP patients with CH were more tended to be initially refractory to corticosteroids. Moreover, ITP patients with CH who failed the corticosteroids were more likely to be resistant to the subsequent treatments. According to the Chinese guidelines, refractory ITP comprises patients who failed the initial treatments and were resistant to rituximab and TPO-RAs, or splenectomy [8]. The proportion of refractory cases in patients with CH was significantly higher than that in non-CH patients (36.4% vs. 4.5%, *P* = 0.002, Fig. 1N). Therefore, detection of CH in ITP might indicate a poor treatment response.

Targeted gene sequencing could be helpful in the differential diagnosis of ITP [5]. The misdiagnosis rate in CH-harboring patients was higher than in the non-CH group though it did not reach the statistical significance (15.4% vs. 2.2%, *P* = 0.117). It was noteworthy that one patient did not harbor any mutation in the coding regions of the customed 38 genes, whereas she was found to have several mutations when using an expanded sequencing panel (CARD11 26.4%, GATA1 49.0%), and finally diagnosed with congenital thrombocytopenia. This suggested a role for whole-genome-sequencing in refractory ITP. Therefore, suspected ITP patients with specific mutations, especially those who responded poorly to the initial treatments, should be followed closely for the possible misdiagnosis and development of myeloid malignancies.

Taking together, CH happened at a low incidence in ITP. It was associated with bleeding severity and responsiveness to current therapies. To our knowledge, this is the first study to evaluate the profile of CH in ITP. Despite the relatively small sample size and short follow-up period, our data suggested that targeted gene sequencing might be helpful in the differential diagnosis of thrombocytopenia. Further investigation is warranted to assess the effects of mutations in certain specific genes on the development of ITP.

## Supplementary information

CH in ITP-supplemental data

## Data Availability

For original data, please contact Liuxingrant@163.com.
