# Supplementary material for: Clonal hematopoiesis in primary immune thrombocytopenia
Source: Blood Cancer J. 2022 Mar 15;12(3):40. doi: 10.1038/s41408-022-00641-5 (PMC8924250; doi:10.1038/s41408-022-00641-5)
Supplement: Supplementary file 1 — CH in ITP-supplemental data [file 41408_2022_641_MOESM1_ESM.pdf]

Clonal Hematopoiesis in Primary Immune Thrombocytopenia
CH in ITP

Supplemental table 1. 38 targeted genes and the sequencing range

Gene name	Chromosome	Transcription ID	Detection range(including splicing site)
ASXL1	20q11	NM_015338.5	Exon1-13
BCOR	Xp11.4	NM_029510.3	Exon2-15
BCORL1	Xq26.1	NM_178782.4	Exon1-12
CALR	19p13.13	NM_004343.3	Exon9
CBL	11q23.3	NM_005188.3	Exon8-9
CEBPA	19q13.1	NM_004364.4	Exon1
CSF3R	1p34.3	NM_004364.4	Exon14,17
DNMT3A	2p23	NM_022552.4	Exon13-23
ETV6	12p13	NM_001987.4	Exon1-8
EZH2	7q36.1	NM_004456.4	Exon2-20
FLT3	13q12	NM_004119.2	Exon14,15,20
GATA2	3q21.3	NM_032638.4	Exon2-6
IDH1	2q33.3	NM_005896.3	Exon4 c.298_414
IDH2	15q26.1	NM_002168.3	Exon4
JAK2	9p24	NM_004972.3	Exon12,14,16
KIT	4q12	NM_000222.2	Exon14,18,19
KRAS	12p12.1	NM_033360.3	Exon2
MLL	11q23	NM_118540.3	Exon3,7,27
MPL	1p34	NM_005373.2	Exon 10
NPM1	5q35.1	NM_002520.6	Exon 11
NRAS	1p13.2	NM_002524.4	Exon 2-3
PDGFRA	4q12	NM_006206.6	Exon14
PHF6	Xq26.3	NM_032458.2	Exon 2-10
PIGA	Xp22.2	NM_011081.3	Exon1-6
RUNX1	21q22.3	NM_001754.4	Exon 2-9
SETBP1	18q21.1	NM_015559.2	Exon4 c.2453_2682
SF3B1	2q33.1	NM_012433.2	Exon 14-16
SH2B3	12q24	NM_005475.2	Exon2 c.557_732
SRSF2	17q25.1	NM_003016.4	Exon1 c.266_362
TET2	4q24	NM_001127208.2	Exon 3-11
TP53	17p13.1	NM_000546.5	Exon 3-11
U2AF1	21q22.3	NM_006758.2	Exon 2, 6,7,8
WT1	11p13	NM_024426.4	Exon 7-9
ZRSR2	Xp22.2	NM_009453.3	Exon7
NF1	17q11.2	NM_010897.2	Exon29,33
STAG2	Xq25	NM_021465.4	Exon12,28
STAT3	17q21.2	NM_011486.5	Exon21
PPM1D	17q23.2	NM_003620.4	Exon1

Supplemental table 2. Details for the detected mutations in 14 ITP patients

Pt.ID	Ensembl gene	Gene	Mutation	AA change	Transcript	COSMIC	VAF
ITP 1	ENSG00000119772.16	DNMT3A	23:c.G2645A	R882H	ENST00000264709.7	COSV53036153	0.0466
ITP2	ENSG00000171456.17	ASXL1	12:c.1900_1922del	E635fs	ENST00000375687.9	COSG65503	0.0539
ITP 3	ENSG00000119772.16	DNMT3A	23:c.G2645A	R882H	ENST00000264709.7	COSV53036153	0.0454
ITP 4	ENSG00000119772.16	DNMT3A	13:c.1516_1517insGGGGT	H506fs	ENST00000264709.7	COSV53036153	0.0692
ITP 5	ENSG00000141510.17	TP53	7:c.C742T	R248W	ENST00000617185	COSV52662035	0.0739
ITP 6	ENSG00000161547.16	SRSF2	1:c.284_298del	95_100del	ENST00000392485.2	COSG6610	0.0588
ITP 7	ENSG00000171456.17	ASXL1	12:c.1927dupG	G642fs	ENST00000375687.9	COSG497249	0.0945
ITP 8	ENSG00000119772.16	DNMT3A	19:c.C2206T	R736C	ENST00000264709.7	COSV53038112	0.0290
ITP 9	ENSG00000168769.13	TET2	3:c.1065dupT	G355fs	ENST00000380013.8	COSG77298	0.0625
ITP 10	ENSG00000119772.16	DNMT3A	15:c.C1792T	R598*	ENST00000264709.7	COSV53039812	0.0313
ITP 11	ENSG00000119772.16	DNMT3A	16:c.C1903T	R635W	ENST00000264709.7	COSV53038925	0.0540
	ENSG00000168769.13	TET2	11:c.A5603G	H1868R	ENST00000380013.8	COSG77298	0.0580
ITP 12	ENSG00000245848.3	CEBPA	1:c.190delA	164fs	ENST00000498907.3	COSG6	0.0125
ITP 13	ENSG00000119772.16	DNMT3A	19:c.T2195C	F732S	ENST00000264709.7	COSV53048945	0.0171
ITP 14	ENSG00000115524.15	SF3B1	15:c.A2098G	K700E	ENST00000335508.10	COSG68561	0.0129

All annotations and genomic coordinates were based on the Genome Reference Consortium Human Genome Build 38 (GRCh38) reference genome. The mutations were shown in HGVS format. The canonical transcript or for the transcript that had harmful foreboding for the protein structure was revealed in the amino-acid change (AA change). The transcripts with the longest CCDS translation were canonical transcripts. The amino-acid changes were also included in the table.

Abbreviations: Pt. ID, patient identifier; ITP, primary immune thrombocytopenia; AA, amino acid; VAF, variant allele frequency.

Supplemental table 3. Clinical characteristics of 102 ITP patients

	Count (n)	Percentage (%)	CH (n)	No CH (n)	*P* value
**Gender**					
female	56	54.9	6	50	0.999
male	46	45.1	5	41	
**Autoantibody**					
positive	24	23.5	3	21	0.999
negative	78	76.5	8	70	
**Smoking**					
smoker	30	28.4	4	26	0.792
non-smoker	72	71.6	7	65	
**Hypertension**					
yes	20	19.6	1	19	0.413
no	82	80.4	10	72	
**Diabetes**					
yes	17	16.7	1	16	0.543
no	85	83.3	10	75	
**CHD**					
yes	9	8.8	2	7	0.687
no	93	91.2	9	84	
**Cerebral infarction**					
yes	6	5.9	1	5	0.567
no	96	94.1	10	86	

Binary-logistic regression was used to analyze the correlation between various risk factors and the incidence of CH. There was no significant association between CH and gender, smoking, existence of anti-glycoprotein (GP) autoantibody (GPIIb/IIIa or GPIb/IX), hypertension, diabetes, coronary heart disease, or cerebral infarction in ITP patients.

Abbreviations: CH, clonal hematopoiesis; CHD, coronary heart disease.

Supplemental table 4. Blood cell indices of 102 ITP patients

	CH	No CH	*P* value
**WBC (×10^3/mL)**	6.55 ± 3.13	6.95 ± 2.63	0.67
**Neutrophils (×10^3/mL)**	4.48 ± 2.53	4.71 ± 2.46	0.79
**Lymphocytes (×10^3/mL)**	1.46 ± 1.03	1.68 ± 0.71	0.41
**Monocytes (×10^3/mL)**	0.52 ± 0.19	0.47 ± 0.24	0.55
**RBC (×10^6/mL)**	4.08 ± 0.92	4.28 ± 0.79	0.48
**Hemoglobulin (g/L)**	122.78 ± 27.35	122.72 ± 31.95	0.99
**RDW (%)**	15.07 ± 2.97	13.83 ± 1.61	0.17
**MCV (fL)**	93.53 ± 8.68	90.05 ± 7.11	0.25
**HCT (%)**	37.78 ± 7.66	38.40 ± 6.63	0.79
**Platelet (×10^3/mL)**	9.82 ± 7.77	18.07 ± 6.04	0.09

Data were presented as mean ± SD.

Abbreviations: WBC, white blood cell; RBC, red blood cell; RDW, red-cell distribution width; MCV, mean corpuscular volume; HCT, hematocrit.

Supplemental table 5. Details of the mutations in 24 CH-AA patients

Patient ID	Gender	Age (Y)	Gene	Mutation	VAF (%)
1	F	50	MLL	c. G6632A: p. R2211Q	17.17
2	F	53	DNMT3A	c. C2311T: p. R771*	22.90
3	M	55	ASXL1	c. T2400G: p. D800E	19.36
4	M	62	TET2	c. 1836delT: p. P612fs	2.59
5	F	66	DNMT3A	c. C1554+1G > A	3.93
6	M	49	DNMT3A	c. T2578C: p. W860R	10.37
7	M	34	TET2	c. G2862A: p. W954*	4.56
8	M	71	U2AF1	c. C101T: p. S34F	10.52
9	M	57	ASXL1	c. 1927dupG: p. G642fs	14.29
			SETBP1	c. G2602A: p. P868N	7.06
10	M	38	BCOR	c. T1260C: p. D420D	5.04
11	F	81	U2AF1	c. C101T: p. S34F	3.06
12	M	86	DNMT3A	c. 2196delT: p. F732fs	21.15
13	M	43	ASXL1	c. C2077T: p. R693*	13.78
14	M	63	BCOR	c. G4691 A: p. W1564*	8.00
			PIGA	c. G548T: p. C183F	9.30
15	M	65	STAT3	c. A1840C: p.S614R	4.20
			PHF6	c. A831T: p. R277S	3.50
16	F	73	SRSF2	c. C284A: p. P95H	3.30
17	F	42	SF3B1	c. A2098G: p. K700E	30.24
18	F	66	TET2	c. T2096A: p. L699*	10.01
19	M	66	ASXL1	c. 1927_1928del: p. G643fs	20.13
			ETV6	c. C1032A: p. Y344*	20.81
20	M	64	TET2	c. G5541A: p. W1847*	24.16
21	F	27	PIGA	c. 172delC: p. Q58S*fs*3	5.60
22	M	52	BCOR	c. 472dupA: p. S158Kfs*28	23.00
23	M	53	ZRSR2	c. 127dupT: p. S43Ffs*12	17.00
24	F	21	KMT2D	c. C1981T : p. R661C	4.60

Supplemental table 6. Summary of gene mutations in 55 CH-hMDS patients

Gene	Patient number	Mutations	Percent in CH patients	Percent in all mutations
DNMT3A	10	G > A(1); A > G(3);<br>C > T(1); del(2); dup(2); delins(1)	18.18%	13.89%
ASXL1	10	del(3); dup(4); C > T(2); C > A(1)	18.18%	13.89%
SF3B1	10	G > A(1); T > G(1); A > G(6); C > T(2)	18.18%	13.89%
U2AF1	6	C > T(3); C > A(2); A > G(1)	10.91%	8.33%
TET2	5	del(3); dup(1); A > G(1)	9.09%	6.94%
TP53	4	C > A(1); G > A(2); T > C(1)	7.27%	5.56%
IDH2	3	G > A(3)	5.45%	4.17%
RUNX1	3	del(1); dup(1); T > A(1)	5.45%	4.17%
SETBP1	3	G > A(3)	5.45%	4.17%
SRSF2	2	c. C283G: p. P95A<br>c. C284G: p. P95L	3.64%	2.78%
WT1	2	c. 297_298del: p. P100fs<br>c. 593_597dup: p. M200fs	3.64%	2.78%
STAT3	2	c.G1981T: p. D661Y<br>c. C1842G: p. S614R	3.64%	2.78%
ETV6	2	c. 306delT: p. R103fs<br>c. 145delC: p. R49fs	3.64%	2.78%
STAG2	2	c. C646T: p. R216*<br>c. A3025G: p. K1009E	3.64%	2.78%
NPM1	1	c. 859_860insTCTG: p. L287fs	1.82%	1.39%
ZRSR2	1	c. 827+1G > A	1.82%	1.39%
PPM1D	1	c. C1654T: p. R552*	1.82%	1.39%
PHF6	1	c. 27dupA: p. G10fs	1.82%	1.39%
NRAS	1	c. 31_35delinsCGGGA:<br>p. A11_G12delinsRD	1.82%	1.39%
NF1	1	c. 2033dupG: p. I679fs	1.82%	1.39%
NPM1	1	c. 859_860insTCTG: p. L287fs	1.82%	1.39%
CBL	1	c. C1019A: p. P340H	1.82%	1.39%
IDH1	1	c. G395A: p. R132H	1.82%	1.39%
BCOR	1	c. 932delA: p. K311fs	1.82%	1.39%
EZH2	1	c. 1217_1220del: p. K406fs	1.82%	1.39%

In the “mutations” column, the number in parentheses represents the number of patients with this type of mutations. The symbol “ > ” means base substitution.

Abbreviations: dup, duplication; del, deletion; delins, deletion-insertion.
